# Phase equilibrium in Mg-Cu-Y

**DOI:** 10.1038/srep03033

**Published:** 2013-10-23

**Authors:** Mohammad Mezbahul-Islam, Mamoun Medraj

**Affiliations:** 1 Department of Mechanical Engineering, Concordia University, 1455 de Maisonneuve Blvd West, Montreal, Quebec, Canada, H3G 1M8

## Abstract

Magnesium-based bulk metallic glasses (BMG) have potential in applications ranging from biomedical to sports equipment and the Mg-Cu-Y system offers some of the most promising alloys. Phase relations and ternary solubility of the binary and ternary compounds of this system have been experimentally investigated. The Isothermal section of Mg-Cu-Y system at 673 K for the entire composition range has been constructed. Phase relations in the Cu-rich (>66 at.% Cu) region of the Mg-Cu-Y system has been determined for the first time. The homogeneity range of three ternary compounds has been determined. Solidifications behavior of several key alloys have been discussed based on the differential scanning calorimetry (DSC) experiments and thermodynamic calculations. Extensive analysis of the DSC curves has been carried out to relate them to the corresponding phase transformation reactions and temperatures. Some of the most promising metallic glass forming regions have been analyzed using thermodynamic calculations.

Mg-based bulk metallic glass (BMG) formers are very attractive because of their high strength-to-weight ratio^1^. Among them, Mg-Cu-Y has the largest super-cooled liquid region^1^,^2^ which is a very important requirement for the BMG forming ability. But one of the primary difficulties in the formation of metallic glass in the bulk form is its requirement for extremely high cooling rate. This issue could be resolved to some extent using the phenomenon of composition dependency of metallic glass. Ma and co-workers^3^,^4^ have demonstrated that composition differences as small as ~1 wt.% can result in significant changes in the glass forming ability. Therefore, it is possible to reduce the cooling rate significantly and obtain bulk metallic glass by selecting proper composition. Hence, many investigations have been carried out to obtain the best composition of metallic glass in the Mg-Cu-Y system^1^,^2^,^5^,^6^,^7^. Several research works have also been published concerning the preparation, characteristics and prediction of metallic glass^8^,^9^,^10^,^11^,^12^,^13^.

Kim et al.^12^ used driving forces of formation for crystalline phases in order to predict the composition with the highest glass forming ability. Hojvat de Tendler et al.^8^ used extended Miedema's model for the thermodynamic characterization of glass formation. They suggested region of compositions where glass formation is possible by quenching or casting. It was found that most of the fully amorphous alloys in this system are basically Mg-Cu alloys with 5 to 10 at.% Y. Based on the literature study^2^,^5^,^6^,^14^ the most promising glass forming regions are surrounded by blue dotted line in figure 1.

Palumbo and Battezzati^15^ used CALPHAD approach^16^ to describe thermodynamically short range order in the liquid, glass transition behavior and the rapid solidification behavior. However, because of lack of broader understanding of the phase equilibria, their work was not conclusive. Considering the importance of this system and the need for the equilibrium phase relations, thermodynamic modeling of this system was published by Mezbahul-Islam and Medraj^17^ using all the available information from the literature until 2008. However, two recent publications by De Negri et al.^18^ and Solokha et al.^19^ reported the presence of nine additional ternary compounds in the Mg-Cu-Y system. De Negri et al.^18^ also reported an isothermal section at 673 K in the 0–66.7 at.% Cu. The available crystal structure information^18^,^19^,^20^,^21^ of the ternary compounds can be found in the Supplementary Table S1 online.

However, complete understanding of this system is still unknown. The isothermal section of the Mg-Cu-Y system at 673 K with more than 66 at.% Cu has not been confirmed yet. Some of the amorphous alloys have been reported^14^ in the Cu-rich region which is shown by the blue triangle in figure 1. Hence, experimental study is required on this portion (more than 66 at.% Cu) of the phase diagram. Also, very little information about the liquidus surface is available. It is important to understand the solidification behavior and phase transformation temperatures especially for compositions important for metallic glass. A combined approach of thermodynamic calculations and DSC measurements has provided a comprehensive understanding of the phase equilibria and phase transformations in the Mg-Cu-Y system in the present work.

## Results

### Isothermal section at 673 K

Fifteen key samples have been studied in this work to construct the isothermal section of the Mg-Cu-Y system at 673 K. The WDS analysis of the alloys is presented in Table 1. BSE images of some of the alloys (2, 3, 4, 6, 7 and 15) are shown in figure 2. The phase relations in the central portion of the Mg-Cu-Y system were shown by dotted lines in the work of De Negri et al.^18^ because the alloys were not in complete equilibrium even after annealing for four weeks. To resolve this, several alloys (3–6) have been prepared in the current work and annealed for 6 weeks at 673 K. The BSE image of sample 6 (53.2/16.6/30.2 Mg/Cu/Y at.%) is shown in figure 2(d). Still the sample is not in complete equilibrium and showing four phases. However, it was found that τ_3_ (white) always remains within τ_4_ (grey) and never been in contact with τ_6_. This is a typical behavior for peritectic type reaction where τ_3_ decompose to τ_4_ and Mg_2_Y(δ). But complete decomposition of τ_3_ will need very long annealing which was not possible to accomplish in this alloy as oxidation starts after 6 weeks. The non-equilibrium effect is more pronounced in this alloy because τ_3_ is a higher melting temperature compound with a larger solidification region than τ_4_. It reflects the high thermal stability of τ_3_ and its sluggish decomposition kinetics. Therefore, it is decided that τ_6_ has a phase triangulation with τ_4_ and Mg_2_Y(δ) and it is not in equilibrium with τ_3_.

Ternary solubility of all the binary compounds in the Cu-rich region has been identified. The Cu_2_Y, Cu_7_Y_2_, Cu_4_Y and Cu_6_Y compounds dissolve approximately 1 at.% Mg. Although in general the error of the WDS measurement is about ±1 at.% it was found that for these Cu-rich (10–15) alloys, the WDS measurement is more accurate with an error of about ±0.8 at.%. Therefore, it is decided to consider a small amount of Mg solubility (~1 at.%) in these Cu-Y binary compounds. No solubility of Y could be found in the Cu-fcc phase. However, Cu dissolves about 2 to 3 at.% Mg at 673 K which is in good agreement with the results of Rogel'berg^22^ who reported ~3.5 at.% Mg solubility in the Cu-fcc phase. The solubility of Cu_6_Y has been found to be about 87 to 89 at.% Cu in sample 12 (1.1/93.7/5.2 Mg/Cu/Y at.%) which is consistent with the values 85 to 87 at.% Cu reported by Fries et al.^23^ for the binary Cu-Y system. Two three-phase regions MgCu_2_ + τ_1_ + Cu-fcc and τ_1_ + Cu_6_Y + Cu-fcc have been determined. Also, three two-phase regions Cu_2_Y + Cu_7_Y_2_, Cu_4_Y + τ_1_ and Cu_6_Y + Cu-fcc have been identified.

The solubility of Cu in MgY(γ) and Mg_2_Y(δ) has been found to be about 1 at.% which is within the error limits of WDS measurement. However, it is decided to accept this value since De Negri et al.^18^, also reported the same amount of Cu solubility in MgY(γ), Mg_2_Y(δ) and Mg_24_Y_5_(ϵ). The solubility of Y in MgCu_2_ has been found to be about 6 at.%. Three of the ternary compounds τ_2_, τ_3_ and τ_11_ have been found to have solubility ranges. τ_2_ has been found with a homogeneity range ~16.6–22.3 at.% Mg, 61.6–69.4 at.% Cu, and 13.4–16.3 at.% Y. For τ_3_, the solubility has been found to be from ~21.9–18.4 at.% Mg, 38.4–40.7 at.% Cu and 38.2–41.5 at.% Y. De Negri et al.^18^ reported the solubility of τ_11_ to be ~81 to 90 at.% Mg. Three key alloys (3–5) have been prepared in this region and all of them showed that the solubility is from ~84 at.% Mg which is in agreement with De Negri et al.^18^. The upper solubility limit of 90 at.% Mg has been adopted from De Negri et al.^18^.

**Figure 1:**
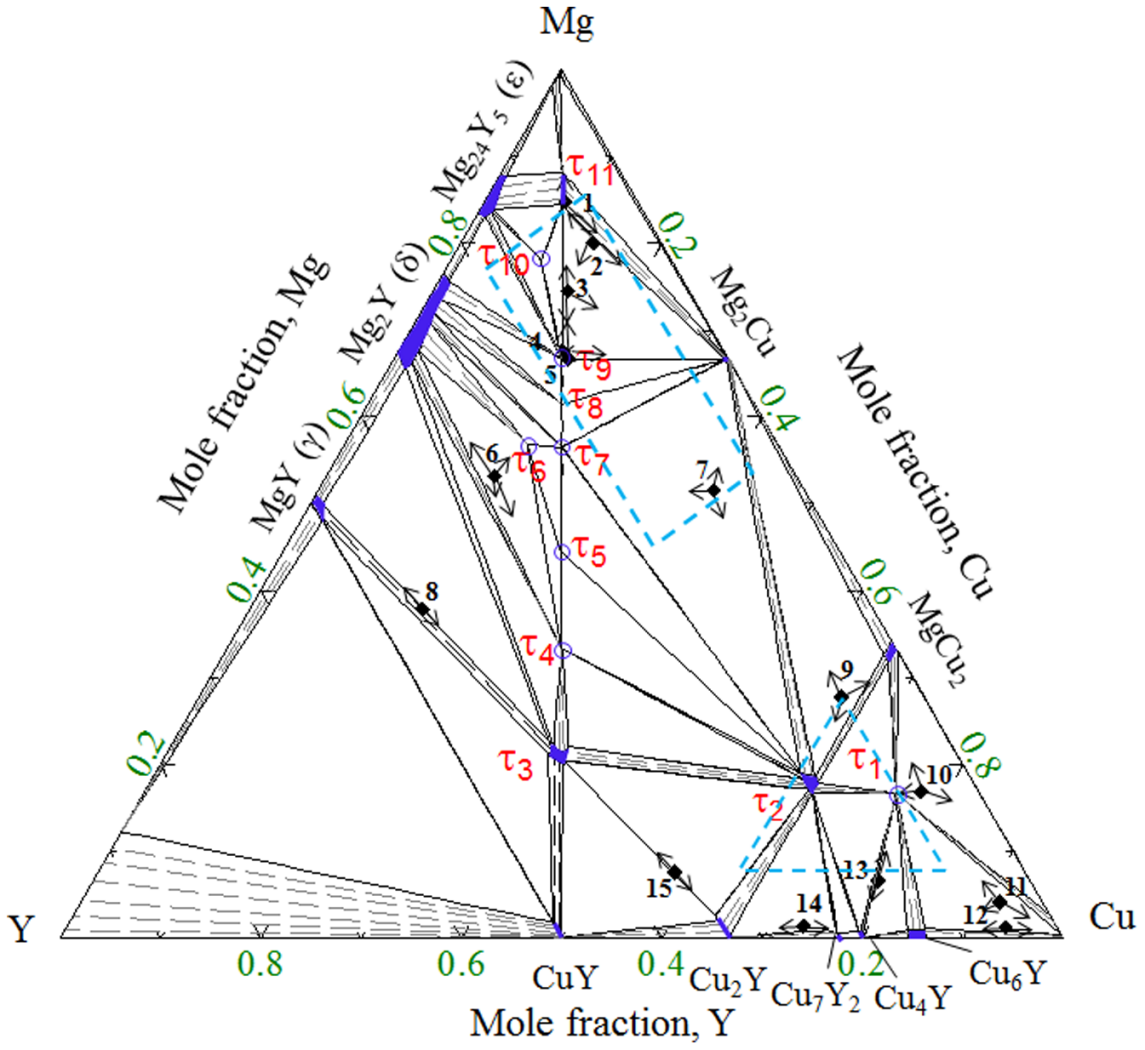
Isothermal section of the Mg-Cu-Y system at 673 K calculated in this work. The solubility of the binary and ternary compounds is shown by dark blue shades. The symbol (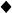) shows the location of the key alloys with the arrow heads pointing towards the phase composition determined by the WDS measurements. The ternary intermetallic compounds are numbered as τ_1_ to τ_11_. The promising metallic glass forming regions are surrounded by blue dotted line.

The phase equilibria of the Mg-Cu-Y system has been understood by combining the analysis of the key alloys from the present work with those reported by De Negri et al.^18^. The thermodynamic modeling of the Mg-Cu-Y system has been modified based on the current understanding of the phase equilibria. During modeling, the present DSC measurements have been used to optimize the thermodynamic model parameters. The isothermal section at 673 K for the whole composition range calculated in this work is shown in figure 1. The phase relations obtained in the present calculations reproduce all the experimental results from the present work as well as from the literature^18^.

### Solidification behavior of the Mg-Cu-Y system

The Mg-Cu-Y is an important metallic glass system. Therefore, it is very important to understand the phase transformations to comprehend the glass forming ability of this system. In this section some of the most promising regions are analyzed in light of the new experiments and thermodynamic calculations. It was observed that most of the glass forming alloys of the Mg-Cu-Y systems contain about 0 to 15 at.% Y^1^,^2^,^6^,^8^,^13^. Therefore, it is decided to explore this region by performing DSC, XRD and thermodynamic calculations on selected key samples to understand the solidification behavior.

### Analysis of the Mg_2_Cu + τ_11_ phase field

Sample 1 (84.9/7.9/7.2 Mg/Cu/Y at.%) is located in the two-phase region of Mg_2_Cu + τ_11_. The phase constituents are summarized in Table 1. Negligible solubility of Y (0.31 ± 1 at.%) has been found in Mg_2_Cu. DSC spectra of this alloy are shown in figure 3. It shows three peaks during heating and three peaks during cooling. However, during cooling the 1^st^ and 3^rd^ peaks show shoulders. Similar results were observed in all the three heating and cooling cycles indicating that some of the peaks overlapped. The thermal arrest points observed during cooling are at temperatures of 774, 751 and 689 K. While during heating the peak temperatures are 752, 708 and 695 K. The first two peaks during cooling were very close and they overlapped during heating cycle and were not distinguishable. The reason for this can be seen in the vertical section corresponding to the sample composition in figure 3. This figure shows that two phase transformations [L/L + hcp-Mg and L + hcp-Mg/L + τ_11_] occur within a narrow temperature range of less than 5 K (from 758 to 755 K). Therefore, during heating these two peaks overlapped with the adjacent dominating peak. Also, areas under the curve between the first two cooling peaks (−144 J/g) and the first heating peak (154 J/g) are similar which confirms that the heating peak is in fact two overlapping peaks. Similar overlapping has been observed for the 3^rd^ peak in the cooling cycle. This is because of another two very close phase transformations [L + τ_11_/L + τ_11_ + Mg_2_Cu and L + τ_11_ + Mg_2_Cu/τ_11_ + Mg_2_Cu] in this region as can be seen in figure 3.

### Analysis of the Mg_2_Cu + τ_9_ + τ_11_ phase field

It has been observed from the literature survey that many promising glass forming alloys lie in the three-phase region of Mg_2_Cu + τ_9_ + τ_11_. Therefore, 4 key samples (2–5) have been prepared in this 3-phase region in order to obtain better understanding of the solidification behavior as well as the phase relationships.

**Figure 2:**
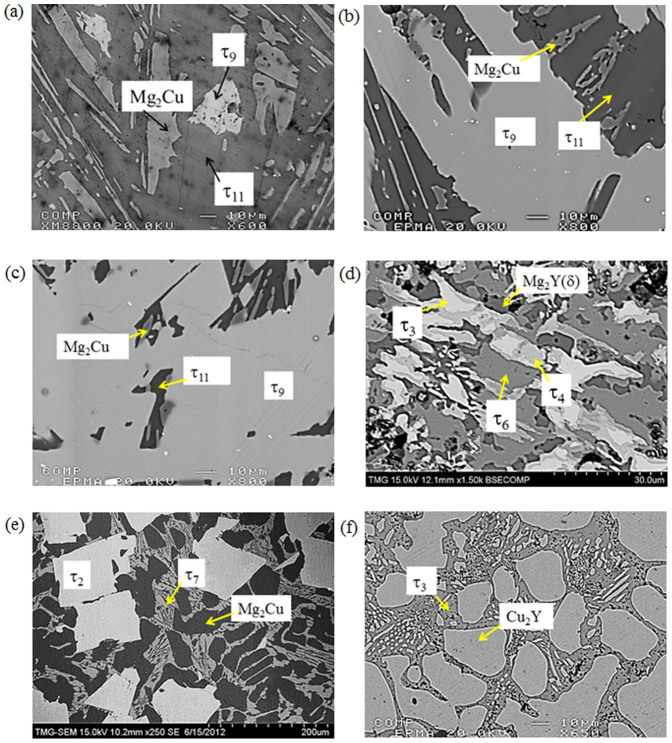
BSE images of selected Mg-Cu-Y alloys; (a) sample 2 (80.4/12.8/6.8 Mg/Cu/Y at.%); (b) sample 3 (70.5/15.3/14.2 Mg/Cu/Y at.%); (c) sample 4 (67.5/16.4/16.1 Mg/Cu/Y at.%); (d) sample 6 (53.2/16.6/30.2 Mg/Cu/Y at.%); (e) sample 7 (55.6/36.9/7.5 Mg/Cu/Y at.%); (f) sample 15 (7.5/57.4/35.1 Mg/Cu/Y at.%).

**Figure 3:**
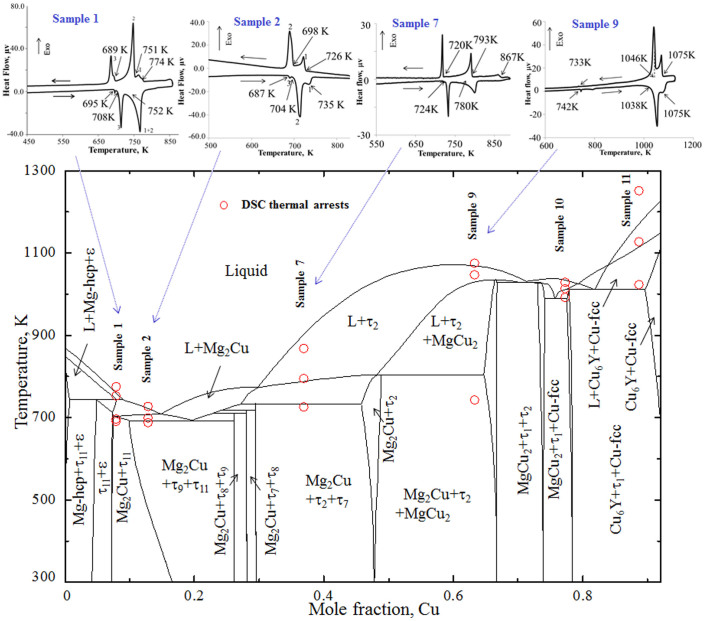
A vertical section calculated at constant 7.2 at.% Y with DSC thermal arrests of samples 1, 2, 7, 9 and 10. The solid phase boundary lines are obtained from the present thermodynamic calculation. The DSC heating and cooling spectra of samples 1, 2, 7 and 9 are shown above. The DSC signals are consistent with the calculated phase transformation temperatures. A deep eutectic region can be seen near 20 at.% Cu. Alloy compositions near this region should be good candidate for metallic glass.

The BSE image of sample 2 (80.4/12.8/6.8 Mg/Cu/Y at.%) in figure 2(a) shows the three-phase equilibrium between Mg_2_Cu, τ_11_ and τ_9_. According to the WDS analysis as listed in Table 1, the matrix is τ_11_ which constitutes 84.7 at.% Mg, 7.1 at.% Cu and 8.2 at.% Y. Since the alloy is very close to τ_11_, large amount of this phase formed in the microstructure. The grey phase is Mg_2_Cu. Small amount of the white phase which is another ternary compound τ_9_, constitutes 68.6 at.% Mg, 16.1 at.% Cu and 15.3 at.% Y. The DSC spectra of this alloy are shown in figure 3. Two exotherms appear in the cooling curve at 726 and 698 K, which correspond to the endotherms that appear in the heating spectrum at 735 and 704 K. Another endothermic signal was revealed in the heating curve at 687 K, but could not be identified in the cooling spectrum. This is because of the supercooling effect which leads to the overlapping of two adjacent cooling peaks. The liquidus temperature of this sample should be in between 726 and 735 K. The calculated vertical section in figure 3 shows good agreement with the DSC signals. The measured thermal arrests of sample 2 correspond to the following phase transformations in the vertical section: L/L + τ_11_/L + τ_11_ + Mg_2_Cu/τ_11_ + Mg_2_Cu + τ_9_. A detailed comparison between the DSC results and the thermodynamic calculations is presented in Table 2.

**Figure 4:**
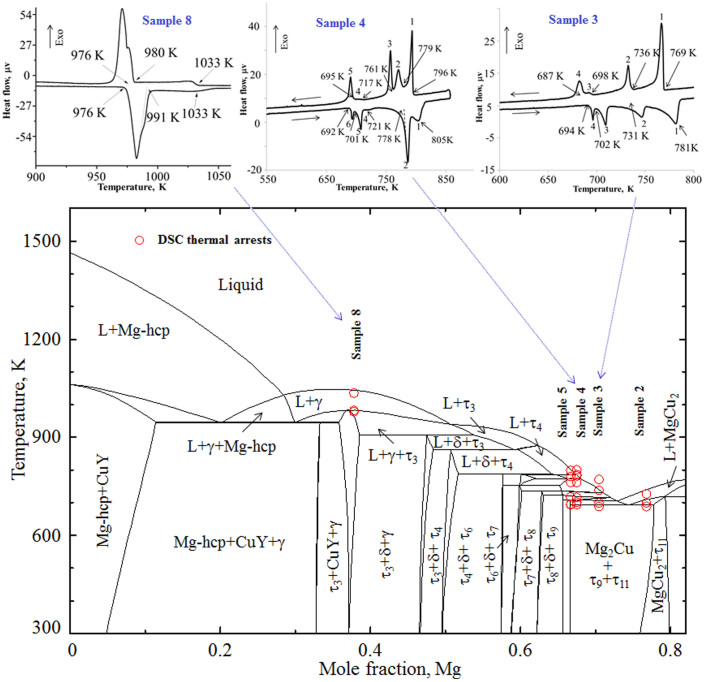
A vertical section calculated at constant 17.2 at.% Cu with DSC thermal arrests of samples 2, 3, 4, 5 and 8. The solid phase boundary lines are obtained from the present thermodynamic calculation. The DSC heating and cooling spectra of samples 3, 4 and 8 are also shown above. The DSC signals are consistent with the calculated phase transformation temperatures.

The next sample in this three-phase field is sample 3 (70.5/15.3/14.2 Mg/Cu/Y at.%). The BSE image, in figure 2(a) clearly shows the existence of the two ternary compounds τ_9_ and τ_11_ along with Mg_2_Cu. The DSC spectra of this alloy are shown in figure 4. Four thermal events during heating as well as cooling are identified. The liquidus temperature is found at 781 K during heating and 769 K during cooling. The thermal arrests are projected on the vertical section in figure 4 which illustrates reasonable agreement. According to the thermodynamic calculation the liquidus temperature is 752 K at which temperature the precipitation of the τ_7_ phase starts. The next thermal arrest is due to the reaction: L + τ_7_/L + τ_8_, which occurs at 734 K compared to the DSC signal at 736 K. Later τ_8_ dissolves more Mg to obtain the stable τ_9_ phase according to L + τ_8_/L + τ_9_ reaction. Thermodynamic calculation shows that this transformation takes place at 721 K. But a clear thermal peak for this reaction could not be identified in the DSC spectra. However, the 2nd thermal event shows a long tail which is probably due to the overlapping of two consecutive peaks and was not separable.

The last two samples (4 and 5) in this phase field are even closer to the ternary compound τ_9_. BSE image of sample 4 (67.5/16.4/16.1 Mg/Cu/Y at.%) in figure 1(c) clearly shows the three phases with massive amount of τ_9_. The growth of τ_9_ with the decrease of Mg content from 70.5 at.% to about 67 at.% can be understood by comparing this alloy in figure 1(c) with sample 3 in figure 1(b). It can be seen that the amount of Mg_2_Cu remains almost the same in these alloys. The amount of τ_9_ increased from ~70% to ~95% in samples 4 and 5 whereas τ_11_ decreased significantly. By comparing these four alloys (2–5), it can be said that for any alloy containing more than ~75 at.% Mg with approximately equal amount of Cu and Y, τ_11_ will be dominant.

The DSC spectra of sample 4 (67.5/16.4/16.1 Mg/Cu/Y at.%) are shown in figure 4. The presence of several thermal events suggests the occurrence of a rather complicated melting behavior. Similar DSC spectra are observed for sample 5 (66.7/17.3/16.0 Mg/Cu/Y at.%) as well. In order to identify the phase transformations more accurately, these two samples have been prepared close to each other. The DSC arrests of samples 4 and 5 are projected on the vertical sections at constant 17.2 at.% Cu as shown in figure 4. The complexity arises because of the presence of six ternary intermetallic compounds (τ_4_ to τ_9_) in close proximity. All these compounds are incongruent and decompose in a narrow temperature range as can be seen in the corresponding vertical section. However, an effort has been made to separate these thermal events according to the equilibrium phase transformation which has been listed in Table 2. The experiments and thermodynamic calculations show reasonable agreement.

One of the criteria for BMG forming alloys is to create chaos where confusion is generated by adding several elements in the alloy to have a sluggish equilibrium^7^,^24^. It can be seen in this vertical section that several phase transformations occur in a relatively narrow temperature range which unsettles the alloys and prevents equilibrium. This slow kinetics can produce a desirable condition for metallic glass.

### Analysis of the Mg_2_Cu + τ_2_ + τ_7_ phase field

Sample 7 (55.6/36.9/7.5 Mg/Cu/Y at.%) is located in the three-phase region of Mg_2_Cu, τ_2_ and τ_7_. The SEM image figure 2(e) clearly shows these three phases. These phases have also been confirmed using XRD analysis. The solubility of Mg in τ_2_ has been found to be ~22.3 at.% which is close to that of De Negri et al.^18^ who reported ~24.0 at.%. The dark matrix in the microstructure is Mg_2_Cu which has negligible Y solubility. The third phase in this sample is τ_7_ which can be seen as a network of the grey phase in the SEM image. The relative weight fractions of the three phases, resulting from Rietveld analysis, are 37% Mg_2_Cu, 24% τ_2_ and 39% τ_7_ which is in qualitative agreement with the microstructure.

The DSC spectra of this alloy are shown in figure 3. Three thermal events are observed and they have been projected on the corresponding vertical section. The predicted phase transformation temperatures are in accord with the DSC measurements. Two thermal arrests are observed at 793 and 720 K during cooling and 780 and 724 K during heating. These two events occurred due to the phase transformation: L + τ_2_/L + τ_2_ + Mg_2_Cu/τ_2_ + Mg_2_Cu + τ_7_. The experimental measurements agree well with the thermodynamic calculations which showed the transformation temperatures at 785 and 731 K, respectively.

### Analysis of the Mg_2_Cu + τ_2_ + MgCu_2_ phase field

**Figure 5:**
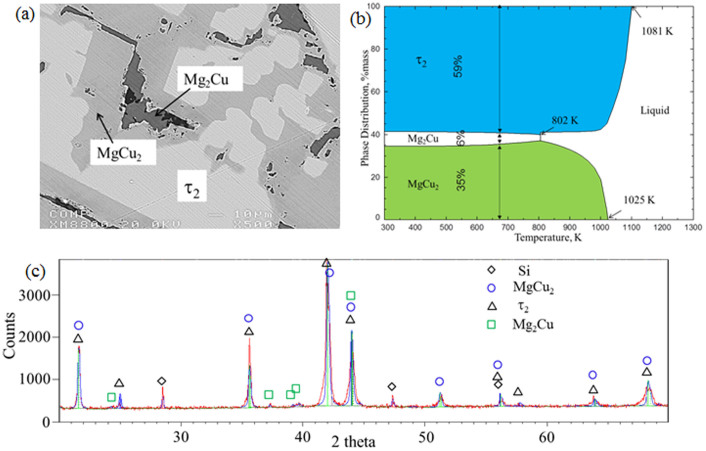
(a) BSE image; (b) phase assemblage diagram; (c) XRD pattern sample 9 (28.0/63.3/8.7 Mg/Cu/Y at.%).

The BSE image of Sample 9 (28.0/63.3/8.7 Mg/Cu/Y at.%) in figure 5 shows the three-phase relationship among MgCu_2_, Mg_2_Cu and τ_2_. The dominating phase is τ_2_ as the sample composition is near to this ternary compound. The DSC spectra in figure 3 show three thermal events during cooling as well as heating. According to the cooling signal the liquidus temperature is 1075 K which agrees well with the thermodynamic calculation of 1081 K where the precipitation of τ_2_ starts. The next transformation occurs at 1046 K (cooling) according to the reaction L + τ_2_/L + τ_2_ + MgCu_2_. The last thermal event is due to the precipitation of the Mg_2_Cu. This signal is very weak because of the small amount of Mg_2_Cu. According to the phase assemblage diagram in figure 5(b) only 6 wt.% of the sample is Mg_2_Cu. The DSC signals have been projected on the vertical section in figure 3 which shows good agreement.

Figure 5(b) shows the phase assemblage diagram of sample 9, where the relative mass versus temperature is calculated. The proportion of each phase at any temperature of interest can easily be interpreted from this figure. For instance, at 673 K, 100 g of the overall material consists of 59 g of τ_2_, 6 g of Mg_2_Cu and 35 g of MgCu_2_. It also shows that while cooling this sample from the melt, τ_2_ solidify first at 1081 K, followed by MgCu_2_ at 1025 K, and then Mg_2_Cu at 802 K. A comparison between the DSC thermal arrest points and the thermodynamic calculations is presented in Table 2. The XRD pattern in figure 5(c) positively identified these three phases. The relative mass fractions of the phases, resulting from Rietveld analysis, are 73 wt.% τ_2_, 5 wt.% Mg_2_Cu and 22 wt.% MgCu_2_ which is in reasonable agreement with the thermodynamic prediction.

### Analysis of the MgCu_2_ + τ_1_ + Cu-fcc phase field

Sample 10 (16.9/77.3/5.8 Mg/Cu/Y at.%) is located in the three-phase region of MgCu_2_ + τ_1_ + Cu-fcc. These phases were detected in the WDS and XRD analyses as listed in Table 1. MgCu_2_ existed in this alloy in two different morphologies having two different concentrations of Y due to the large solubility of MgCu_2_. The alloy undergoes a eutectic reaction. The eutectic structure can be seen in the microstructure (figure S5, supplementary information online). During solidification MgCu_2_ forms first at 1010 K according to the following transformation reaction: L + τ_1_/L + τ_1_ + MgCu_2_. This form of MgCu_2_ dissolved ~5.2 at.% Y. Later due to the eutectic reaction at 985 K MgCu_2_ formed again which was found to dissolve ~1.5 at.% Y. MgCu_2_ has a large ternary solubility which varies with temperature. This is why when it formed at higher temperature it gives different composition than at lower temperature eutectic transformation. In the present work, this indirect information is used to assess the maximum solubility of Y in MgCu_2_. This is in agreement with De Negri et al.^18^ who reported about 5 at.% Y solubility in MgCu_2_ at 673 K. The DSC thermal arrests of this alloy are projected on the vertical section in figure 3 which shows good agreement. Detailed comparison with the thermodynamic predictions is given in Table 2.

### Analysis of the Cu_6_Y + τ_1_ + Cu-fcc phase field

Sample 11 (4.1/91.6/4.3 Mg/Cu/Y at.%) is located in the Cu-rich corner as can be seen in figure 1. It is prepared mainly to establish the phase triangulation in this area since De Negri et al.^18^ did not perform any experiments with more than 66.67 at.% Cu. The WDS and XRD analysis confirmed a three-phase relation of Cu-fcc, Cu_6_Y and τ_1_. Three thermal arrests in heating as well as cooling are detected in the DSC spectra as shown in figure 3. The heating signals: 1256, 1101 and 1022 K correspond well with the cooling signals: 1263, 1126 and 1015 K, respectively. These measured thermal events correspond to the following phase transformations in the vertical section: L/L + Cu-fcc/L + Cu-fcc + Cu_6_Y/τ_1_ + Cu-fcc + Cu_6_Y.

The location of Sample 11 is on the slope of a steep liquidus surface as can be seen in figure 3. This liquid is going towards a deep eutectic and generates a possible glass forming zone. As can be seen in figure 1 some of the fully amorphous alloys are located near this region. According to the current thermodynamic calculations, alloys with 74 to 80 at.% Cu with approximately 5 to 10 at.% Y should be promising candidates for metallic glass.

### Analysis of some important glass forming alloys

**Figure 6:**
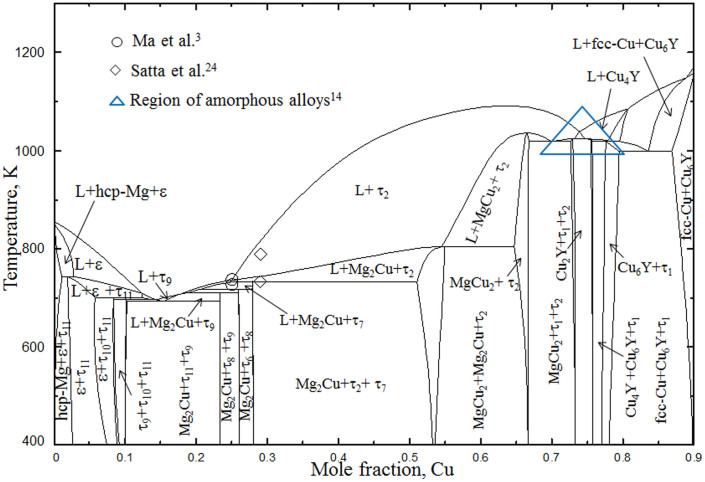
Vertical section at constant 10% Y with DSC results from the literature^3^,^24^.

Inoue et al.^2^ reported Mg_65_Cu_25_Y_10_ to be the most favorable composition for glass formation. They used conventional mold casting and could produce samples up to 4 mm in diameter. They also referred to this composition as eutectic. Later Ma et al.^3^,^10^ reported that they found Mg_58_Cu_30.5_Y_11.5_ and Mg_58.5_Cu_30.5_Y_11.0_ compositions which show higher glass forming ability. They produced fully amorphous samples of upto 9 mm diameter. However, they found these compositions little bit away from the eutectic point. Therefore, they suggested that the optimum glass forming alloys should be found at off-eutectic locations. Satta et al.^24^ investigated the Mg_65_Cu_25_Y_10_ alloy and compared their results with preliminary thermodynamic understanding of the ternary system. To obtain equilibrium state they annealed the amorphous alloy at 713 K for two weeks. But during annealing the sample lost 4 at.% Mg and obtained a final composition of Mg_61_Cu_29_Y_10_. They recognized three different phases in the sample but only could identify Mg_2_Cu in the XRD pattern. According to their EDS analysis these two phases have compositions of 60/23/17 and 65/20/15 Mg/Cu/Y at.%. Based on the current work, these two phases can be identified as τ_7_ and τ_8_, respectively. Both Ma et al.^3^ and Satta et al.^24^ used DSC experiments to identify the liquidus and solidus temperatures of the amorphous alloys. These measurements were done at relatively high heating rate of 20 K/min and mostly on non-equilibrium samples. Also, the reproducibility of these measurements was not confirmed. Therefore these results were not considered during the thermodynamic optimization but are compared with the current calculations. A vertical section at 10 at.% Y is presented in figure 6 with the DSC measurements of Ma et al.^3^ and Satta et al.^24^ which shows good agreement. According to Ma et al.^3^,^10^ the best glass forming alloys should be found at an off-eutectic composition which is not far from the deep eutectic point. One of the ternary eutectic points has been found near 75.3/15.2/9.5 Mg/Cu/Y at.% composition in the present calculation. Considering this and observing the vertical section in figure 6, it can be said that alloys with around 10 at.% Y and 10 to 20 at.% Cu are promising candidate for metallic glass. Some of the metallic glass alloys have been reported^14^ in the Cu-rich region of the Mg-Cu-Y system as shown by the blue triangle in figures 1 and 6. The liquidus or solidus temperature of these alloys could not be found in the literature. However, it can be seen in figure 6 that the liquidus is going towards a eutectic point generating a suitable glass forming region.

## Discussion

A comprehensive investigation of the Mg-Cu-Y system is carried out using XRD, DSC and WDS analyses coupled with thermodynamic calculations. This research provides a much needed bridge between the thermodynamic modeling and experimental work on this system. The isothermal section of the Mg-Cu-Y system at 673 K for the whole composition range is constructed based on the current experiments as well as from the literature. Phase relations with more that 67 at.% Cu are established experimentally for the first time. Approximately 1 at.% Mg solubility is found in Cu_2_Y, Cu_7_Y_2_, Cu_4_Y and Cu_6_Y. No solubility of Y can be found in Cu-fcc. Eleven ternary compounds are identified in the Mg-Cu-Y system. Nine of these compounds are located along the Mg-NiY line. Because of the peritectic decomposition of most of these compounds, the alloys near the central portion of the phase diagram show sluggish kinetics. The homogeneity range of τ_2_, τ_3_ and τ_11_ is determined. Extensive discussion on the interpretation of the DSC spectra is carried out to understand the melting and solidification behavior of the alloys. The phase transformation temperatures detected by the DSC are compared with the pertinent vertical sections. Reasonable agreement between experimental and thermodynamic calculations is observed. The present understanding of the system is also used to explain the solidification behavior of some of the most promising glass forming regions. It has been found that higher glass forming alloys lie on the valley of the deep eutectic and not exactly on the eutectic point. Since metallic glasses are very much composition dependent, the present analysis would be useful to identify more alloys with glass forming ability.

## Methods

### Sample preparation and characterization

Fifteen key alloys were chosen by critical assessment of the experimental data and thermodynamic calculations. Table 1 and figure 1 show the different investigated samples and their compositions. The purity of the elements used is Mg-99.8 wt.%, Cu-99.8 wt.%, and Y-99.9 wt.%. Samples 1 to 10 are prepared at CANMET where first, Cu is melted at 1373 K under protective argon flow. Then Y is added and the temperature is raised above the liquidus. The melt is held at this temperature until Y dissolves completely. The furnace temperature is then lowered below 1173 K and Mg is plunged. The melt is stirred slowly with graphite rod and let to solidify in the crucible. Key samples 11 to 15 are prepared at Concordia University using an arc melting furnace with water cooled copper crucible. The chamber is evacuated and purged by argon several times before melting. Each alloy is crushed and re-melted at least four times to ensure homogeneity. The actual global compositions of the samples are identified by Inductively Coupled Plasma-Optical Emission Spectrometry (ICP-OES). The key alloys are placed in a tantalum container and sealed in a quartz tube under protective Ar atmosphere. These are then annealed at 673 K for 4 weeks. However, for key samples 3 to 6 annealing for 6 weeks was necessary to obtain equilibrium condition.

The annealed samples are characterized by light optical microscopy (OM), scanning electron microscopy (SEM) and wave dispersive X-ray spectrometer (WDS). The error of the WDS measurements is estimated to be ±1 at.%. The XRD patterns are obtained using PANanalytical Xpert Pro powder X-ray diffractometer with a CuKα radiation. The XRD spectrum is acquired from 20 to 120° with a 0.02° step size. The obtained diffraction patterns are refined and analyzed using X'Pert HighScore Plus Rietveld analysis software.

Thermal investigation is performed using a Setaram Setsys DSC-2400 instrument. Temperature calibration of the DSC is done using standard samples of Pb, Sn, Zn, Ag and Au. The samples are cut and mechanically polished to remove any possible contaminated surface layers. Afterwards, they are cleaned with 99% ethanol and placed in an alumina crucible with a lid cover. To avoid oxidation, evacuations followed by rinses with argon are done. The DSC measurements are carried out under flowing argon atmosphere with the same heating and cooling rate of 5 K/min. The weight of the sample is kept in the range 50 ~ 70 mg. The reproducibility of every measurement is confirmed by collecting the data during three heating and cooling cycles on two different replicas of each sample. The estimated error of measurements between the repetitive cycles is ±7 K. Temperatures corresponding to various thermal events are obtained from the analysis of the DSC curves. According to the general recommendations of Boettinger et al.^25^ for metallic alloys, during heating, peak onset is taken as the phase transformation temperature, while peak maximum is used as the liquidus temperature. While cooling peak onset is used for both phase transformation and liquidus temperature. In this work, the onset of cooling peaks is compared with the thermodynamic calculations except the last transformation to solid for which the onset of heating is considered. The liquidus temperatures of the alloys were not apparent during heating and were difficult to interpret. Also, samples with more than two arrests show pronounced overlapping during heating. However, both the heating and cooling signals are listed in Table 2 and compared with the thermodynamic calculations for better understanding.

### Thermodynamic calculation

Vertical sections and phase assemblage diagrams are drawn using the modified thermodynamic modeling of this system which will be published elsewhere. The vertical section shows the sequence of the thermal events during heating and cooling. The phase assemblage diagram shows the phase transformation temperature during thermal session as well as the relative amount of each phase at any temperature which in turn serves as a guidance to understand the DSC patterns. All the thermodynamic calculations are performed using FactSage 6.4^26^ software.

## Supplementary Material

Supplementary Information Supplementary information

## Figures and Tables

**Table 1:** SEM-WDS and XRD data on selected Mg-Cu-Y alloys annealed at 673 K

Actual Composition				Identified phases using XRD and WDS			
	at.%				Compositions by WDS		
No	Mg	Cu	Y	Name	Mg	Cu	Y
1	84.9	7.9	7.2	Mg_2_Cu	68.9	30.8	0.3
				τ_11_	84.4	7.3	8.3
2	80.4	12.8	6.8	Mg_2_Cu	68.6	31.3	0.1
				τ_11_	84.7	7.1	8.2
				τ_9_	68.6	16.1	15.3
3	70.5	15.3	14.2	Mg_2_Cu	69.6	30.3	0.1
				τ_11_	85.0	7.0	8.0
				τ_9_	68.9	15.9	15.2
4	67.5	16.4	16.1	Mg_2_Cu	69.4	30.4	0.2
				τ_11_	84.0	7.6	8.4
				τ_9_	69.2	15.9	14.9
5	66.7	17.3	16.0	Mg_2_Cu	69.6	30.1	0.3
				τ_11_	84.1	7.7	8.2
				τ_9_	68.9	16.0	15.1
6*	53.2	16.6	30.2	Mg_2_Y(δ)	66.3	0.7	33.0
				τ_7_	53.9	19.8	26.3
				τ_4_	33.1	33.1	33.8
				τ_3_	18.4	40.6	41.0
7	55.6	36.9	7.5	Mg_2_Cu	65.1	34.6	0.3
				τ_2_	22.3	62.5	15.3
				τ_6_	53.5	23.6	23.9
8	37.8	17.2	45.0	MgY(γ)	48.8	1.1	50.1
				τ_3_	19.8	38.7	41.5
9	28.0	63.3	8.7	Mg_2_Cu	67.1	32.8	0.1
				τ_2_	17.7	69.3	13.0
				MgCu_2_	26.9	66.8	6.3
10	16.9	77.3	5.8	Cu-fcc	2.7	97.2	0.1
				τ_1_	17.9	73.8	8.3
				MgCu_2_	33.0–27.0	65.5–71.8	1.5–5.2
11	4.1	91.6	4.3	Cu-fcc	1.9	97.9	0.2
				Cu_6_Y	1.1	86.6	12.3
				τ_1_	14.7	76.9	8.3
12	1.1	93.7	5.2	Cu-fcc	1.4	98.5	0.1
				Cu_6_Y	0.7–0.8	87.4–89.6	11.9–9.6
13	6.7	78.2	15.1	Cu_4_Y	1.8	79.6	18.6
				τ_1_	14.9	76.1	0.9
14	1.4	73.3	25.3	Cu_7_Y_2_	2.0	77.4	20.6
				Cu_2_Y	0.6	65.3	34.1
15	7.5	57.4	35.1	τ_3_	21.9	39.9	38.2
				Cu_2_Y	0.3	66.2	33.5

*Appearance of four phases due to peritectic reaction.

**Table 2:** Phase constituents by XRD and WDS and DSC measurements and calculated transformation temperature of the investigated samples (h denotes heating and c denotes cooling)

			Thermodynamic calculation	
Sample	Identified phases using WDS and XRD	DSC thermal signals, K	Temperature, K	Reaction or phase boundary
1	Mg_2_Cu	774c	758	L/L + hcp-Mg
	τ_11_	751c/752h	755	L + hcp-Mg/L + τ_11_
		689c/708h	708	L + τ_11_/L + Mg_2_Cu + τ_11_
		695h	707	L + Mg_2_Cu + τ_11_/Mg_2_Cu + τ_11_
2	Mg_2_Cu	726c/735h	728	L/L + τ_11_
	τ_11_	698c/704h	706	L + τ_11_/L + τ_11_ + Mg_2_Cu
	τ_9_	687h	699	L + τ_11_ + Mg_2_Cu/τ_11_ + Mg_2_Cu + τ_9_
3	Mg_2_Cu	769c/781h	752	L/L + τ_7_
	τ_11_	736c/731h	734	L + τ_7_/L + τ_8_
	τ_9_	-	721	L + τ_8_/L + τ_9_
		698c/702h	698	L + τ_9_/L + τ_9_ + τ_11_
		687c/694h	692	L + τ_9_ + τ_11_/τ_9_ + τ_11_ + Mg_2_Cu
4	Mg_2_Cu	798c/798h	789	L/L + τ_4_
	τ_11_	784c	784	L + τ_4_/L + τ_5_
	τ_9_	781c	777	L + τ_5_/L + τ_6_ + τ_7_
		761c/772h	765	L + τ_6_ + τ_7_/L + τ_7_
		-	734	L + τ_7_/L + τ_8_
		714c	721	L + τ_8_/L + τ_9_
		700c/707h	696	L + τ_9_/L + τ_9_ + τ_11_
		693h	692	L + τ_9_ + τ_11_/Mg_2_Cu + τ_9_ + τ_11_
5	Mg_2_Cu	796c/805h	801	L/L + τ_4_
	τ_11_	779c/778h	784	L + τ_4_/L + τ_5_
	τ_9_	761c	773	L + τ_5_/L + τ_7_
		-	733	L + τ_7_/L + τ_8_
		717c/721h	720	L + τ_8_/L + τ_8_ + τ_9_
		695c/701h	709	L + τ_8_ + τ_9_/L + τ_9_ + Mg_2_Cu
		692h	692	L + τ_9_ + Mg_2_Cu/Mg_2_Cu + τ_9_ + τ_11_
7	Mg_2_Cu	867c	915	L/L + τ_2_
	τ_2_	793c/780h	785	L + τ_2_/L + τ_2_ + Mg_2_Cu
	τ_7_	720c/724h	732	L + τ_2_ + Mg_2_Cu/τ_2_ + Mg_2_Cu + τ_7_
8	MgY(γ)	1033c/1033h	1043	L/L + γ
	τ_3_	980c/991h	981	L + γ/L + τ_3_ + γ
		976c/976h	963	L + τ_3_ + γ/τ_3_ + γ
9	Mg_2_Cu	1075c/1075h	1081	L/L + τ_2_
	τ_2_	1046c/1038h	1025	L + τ_2_/L + τ_2_ + MgCu_2_
	MgCu_2_	733c/742h	802	L + τ_2_ + MgCu_2_/τ_2_ + MgCu_2_ + Mg_2_Cu
10	Cu-fcc	1028c/1040h	1017	L/L + τ_1_
	τ_1_	1012c/1001h	1010	L + τ_1_/L + τ_1_ + MgCu_2_
	MgCu_2_	989c/991h	985	L + τ_1_ + MgCu_2_/τ_1_ + MgCu_2_ + Cu-fcc
11	Cu-fcc	1263c/1256h	1239	L/L + Cu-fcc
	Cu_6_Y	1126c/1101h	1099	L + Cu-fcc/L + Cu-fcc + Cu_6_Y
	τ_1_	1015c/1022h	1009	L + Cu-fcc + Cu_6_Y/Cu-fcc + Cu_6_Y + τ_1_
15	τ_2_	1119c/1112h	1066	L/L + Cu_2_Y
	Cu_2_Y	1043c	1040	L + Cu_2_Y/L + Cu_2_Y + CuY
		996c/998h	992	L + Cu_2_Y + CuY/L + Cu_2_Y + τ_2_

## References

[b1] Busch R., Liu W. & Johnson W. L. Thermodynamics and kinetics of the Mg65Cu25Y10 bulk metallic glass forming liquid. J. Appl. Phys. 83, 4134–4141 (1998).

[b2] Inoue A., Kato A., Zhang T., Kim S. G. & Masumoto T. Magnesium-copper-yttrium amorphous alloys with high mechanical strengths produced by a metallic mold casting method. Mater. Trans., JIM 32, 609–616 (1991).

[b3] Ma H., Zheng Q., Xu J., Li Y. & Ma E. Doubling the critical size for bulk metallic glass formation in the Mg-Cu-Y ternary system. J. Mater. Res. 20, 2252–2255 (2005).

[b4] Zheng Q., Ma H., Ma E. & Xu J. Mg-Cu-(Y,Nd) pseudo-ternary bulk metallic glasses: The effects of Nd on glass-forming ability and plasticity. Scripta Mater. 55, 541–544 (2006).

[b5] Kim S. G., Inoue A. & Masumoto T. High mechanical strengths of magnesium-nickel-yttrium and magnesium-copper-yttrium amorphous alloys with significant supercooled liquid region. Mater. Trans., JIM 31, 929–934 (1990).

[b6] Murty B. S. & Hono K. Formation of nanocrystalline particles in glassy matrix in melt-spun Mg-Cu-Y based alloys. Mater. Trans., JIM 41, 1538–1544 (2000).

[b7] Katz-Demyanetz A., Rosenson H., Koren Z. & Regev M. Bulk metallic glass formation in the Mg-Cu-Y system. Mater. Sci. Technol. 25, 1227–1233 (2009).

[b8] Hojvat de Tendler R. *et al.* Calculation of metastable free-energy diagrams and glass formation in the Mg–Cu–Y alloy and its boundary binaries using the Miedema model. Intermetallics 14, 297–307 (2006).

[b9] Lu Z. P., Li Y. & Ng S. C. Reduced glass transition temperature and glass forming ability of bulk glass forming alloys. J. Non-Cryst. Solids 270, 103–114 (2000).

[b10] Ma D. & Chang Y. A. Competitive formation of ternary metallic glasses. Acta Mater. 54, 1927–1934 (2006).

[b11] Takeuchi A. & Inoue A. Mixing enthalpy of liquid phase calculated by Miedema's scheme and approximated with sub-regular solution model for assessing forming ability of amorphous and glassy alloys. Intermetallics 18, 1779–1789 (2010).

[b12] Kim D., Lee B.-J. & Kim N. J. Prediction of composition dependency of glass forming ability of Mg-Cu-Y alloys by thermodynamic approach. Scripta Mater. 52, 969–972 (2005).

[b13] Chen G. & Ferry M. Crystallization and thermally induced surface relief effects in a Mg65Cu25Y10 bulk metallic glass. J. Mater. Sci. 42, 646–651 (2007).

[b14] Institute for Materials Research of Tohoku University. KIND Data Base. http://www-db2.imr.tohoku.ac.jp/kind/11_Amor_Ternary/Cu-Mg-Y.html (Cited on October 30 2012).

[b15] Palumbo M. & Battezzati L. Thermodynamics and kinetics of metallic amorphous phases in the framework of the CALPHAD approach. Calphad 32, 295–314 (2008).

[b16] Kaufman L. & Bernstein H. Computer Calculation of Phase Diagrams; With Special Reference to Refractory Metals. (Refractory Materials, Vol. 4), (Academic, 1970).

[b17] Mezbahul-Islam M., Kevorkov D. & Medraj M. The equilibrium phase diagram of the magnesium–copper–yttrium system. J. Chem. Thermodyn. 40, 1064–1076 (2008).

[b18] De Negri S., Solokha P., Saccone A. & Pavlyuk V. The Y–Cu–Mg system in the 0–66.7 at.% Cu concentration range: The isothermal section at 400°C. Intermetallics 17, 614–621 (2009).

[b19] Solokha P., De Negri S., Pavlyuk V. & Saccone A. Inhomogeneous 2D linear intergrowth structures among novel Y–Cu–Mg ternary compounds with yttrium/copper equiatomic ratio. Solid State Sci. 11, 801–811 (2009).

[b20] Solokha P. *et al.* Rare earth–copper–magnesium compounds RECu_9_Mg_2_ (RE=Y, La–Nd, Sm–Ho, Yb) with ordered CeNi_3_-type structure. J. Solid State Chem. 179, 3073–3081 (2006).

[b21] Mishra R., Hoffmann R.-D. & Pottgen R. New magnesium compounds RE_2_Cu_2_Mg (RE=Y, La - Nd, Sm, Gd - Tm, Lu) with Mo_2_FeB_2_ type structure. Z. Naturforsch., B: Chem. Sci. 56, 239–244 (2001).

[b22] Rogel'berg L. Solubility of Mg in Cu and Combined Solubility of Mg and Al in Cu. Tr. Gosud. N.-I. Pr. Inst. Obrab. Tsvetn. Met. (Russian) 16, 82–89 (1957).

[b23] Fries S. G., Lukas H. L., Konetzki R. & Schmid-Fetzer R. Experimental investigation and thermodynamic optimization of the Y-Cu binary system. J. Phase Equilib. 15(**6**), 606–614 (1994).

[b24] Satta M., Palumbo M., Rizzi P. & Baricco M. Ternary compounds and glass formation in the Cu-Mg-Y system. Adv. Eng. Mater. 9, 475–479 (2007).

[b25] Boettinger W. J., Kattner U. R., Moon K. W. & Perepezko J. H. NIST Recommended Practice Guide: DTA and Heat-Flux DSC Measurements of Alloy Melting and Freezing. Vol. Special Publication 960–15 (Elsevier, Kidlington, -1 2006).

[b26] Bale C., Pelton A. & Thompson W. FactSage 6.4. Factsage thermochemical software and databases, http://www.crct.polymtl.ca/. (Cited on October 30 2012).

