# Supplementary material for: Phase equilibrium in Mg-Cu-Y
Source: Sci Rep. 2013 Oct 23;3:3033. doi: 10.1038/srep03033 (PMC6505671; doi:10.1038/srep03033)
Supplement: Supplementary Information [file srep03033-s1.pdf]

PHASE EQUILIBRIUM IN MG-CU-Y

Mohammad Mezbahul-Islam and Mamoun Medraj

Department of Mechanical Engineering, Concordia University,
1455 de Maisonneuve Blvd West, Montreal, Quebec, Montreal, Canada, H3G 1M8

* Corresponding author, email: mmedraj@encs.concordia.ca
Tel: +1 514 848 2424 ext. 3146; Fax: +1 514 848-3175

Supplementary information

Table T.S.1: Crystallographic data on the ternary compounds of the Mg-Cu-Y system

Phase	Pearson's symbol	Structure type	Space group	Lattice parameters, Å			Ref.
				a	b	c	
Mg_2Cu_9Y (τ_1)	hp24	$TbCu_9Mg_2$	$P6_3/mmc$	5.0044		16.2031	[1]
$MgCu_4Y$ (τ_2)	cF24	$MgCu_4Sn$	$F\bar{4}3m$	7.231			[2]
$MgCu_2Y_2$ (τ_3)		Mo_2FeB_2	P6/mbm	7.6265		3.7409	[3]
MgCuY (τ_4)	hp9,3	ZrNiAl	$P\bar{6}2m$	7.4449	3.9953	1.9178	[2]
$Mg_8Cu_5Y_5$ (τ_5)	oP36	$Mg_8Cu_5Y_5$	Pmma	26.3723	4.0066	74.115	[4]
$Mg_{13}Cu_5Y_5$ (τ_6)	oS92,4	$Mg_{13}Cu_5Y_5$	Cmcm	4.0973	19.2794	25.7907	[4]
$Mg_{57}Cu_{18}Y_{25}$ (τ_7)	Not available						
$Mg_{16}Cu_5Y_5$ (τ_8)	oS104,4	$Mg_{16}Cu_5Y_5$	Cmcm	4.1360	19.239	29.086	[4]
Mg_4CuY (τ_9)	oS48,8	$TbCuMg_4$	Cmmm	13.5754	20.3153	3.9060	[4]
$Mg_{78}Cu_9Y_{13}$ τ_{10})	Not available						
$Mg_{9\text{-}18}CuY$ (τ_{11})	Not available						

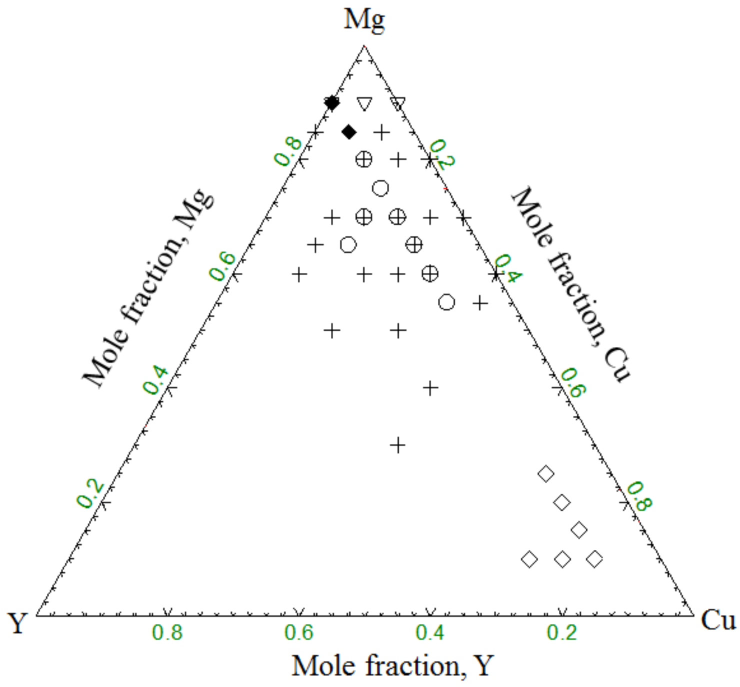

Figure S1: Composition of the published glass forming alloys: (O) amorphous alloys [5]; (♦) crystalline alloys [5]; (+) amorphous alloys [6]; (∇) crystalline alloys [6]; (◊) amorphous alloys [7]. It can be seen that most of the fully amorphous alloys are basically Mg-Cu alloys with 5 to 10 at.% Y. Based on this observation the most promising glass forming regions are shown by the blue dotted line in figure 1.

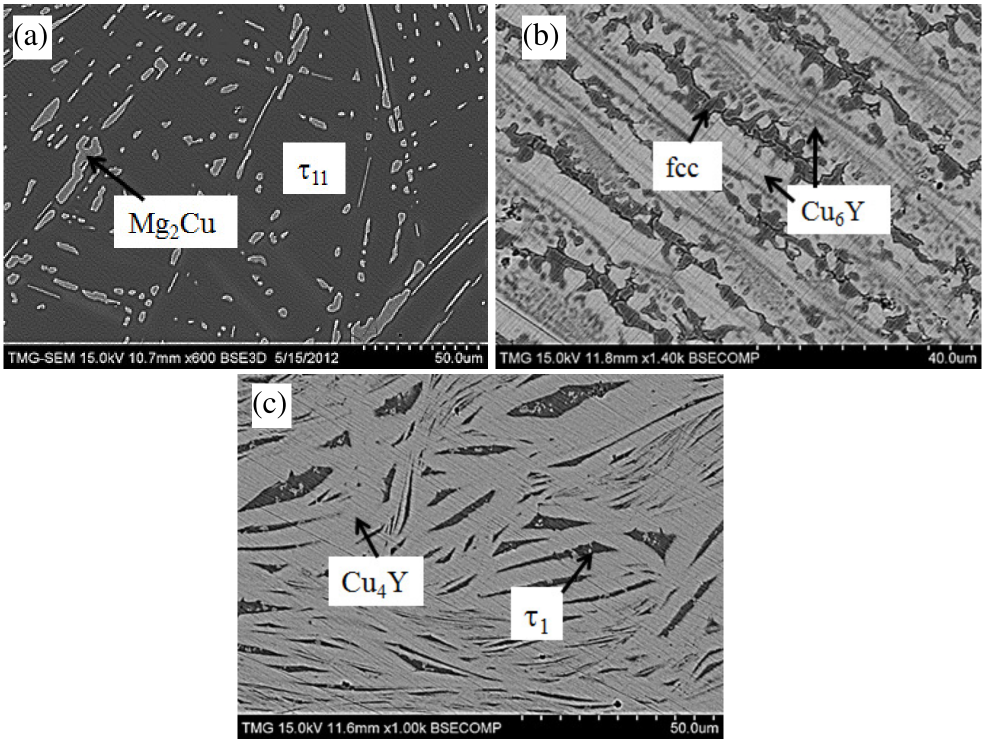

Figure S2: BSE image of (a) sample 1 (84.9/7.9/7.2 Mg/Cu/Y at.%) is showing Mg_2Cu and τ_{11}. In this micrograph the dark matrix is τ_{11} whereas plate like brighter structure is Mg_2Cu; (b) sample 12 (1.1/93.7/5.2 Mg/Cu/Y at.%) is showing the phase relations of Cu-fcc and Cu_6Y. Cu_6Y is found in white and grey shades due to the solid solubility. The homogeneity of this compound has been found to be about 87 to 89 at.% Cu. (b) sample 13 (6.7/78.2/15.1 Mg/Cu/Y at.%) is showing the phase relation of τ_1 and Cu_4Y.

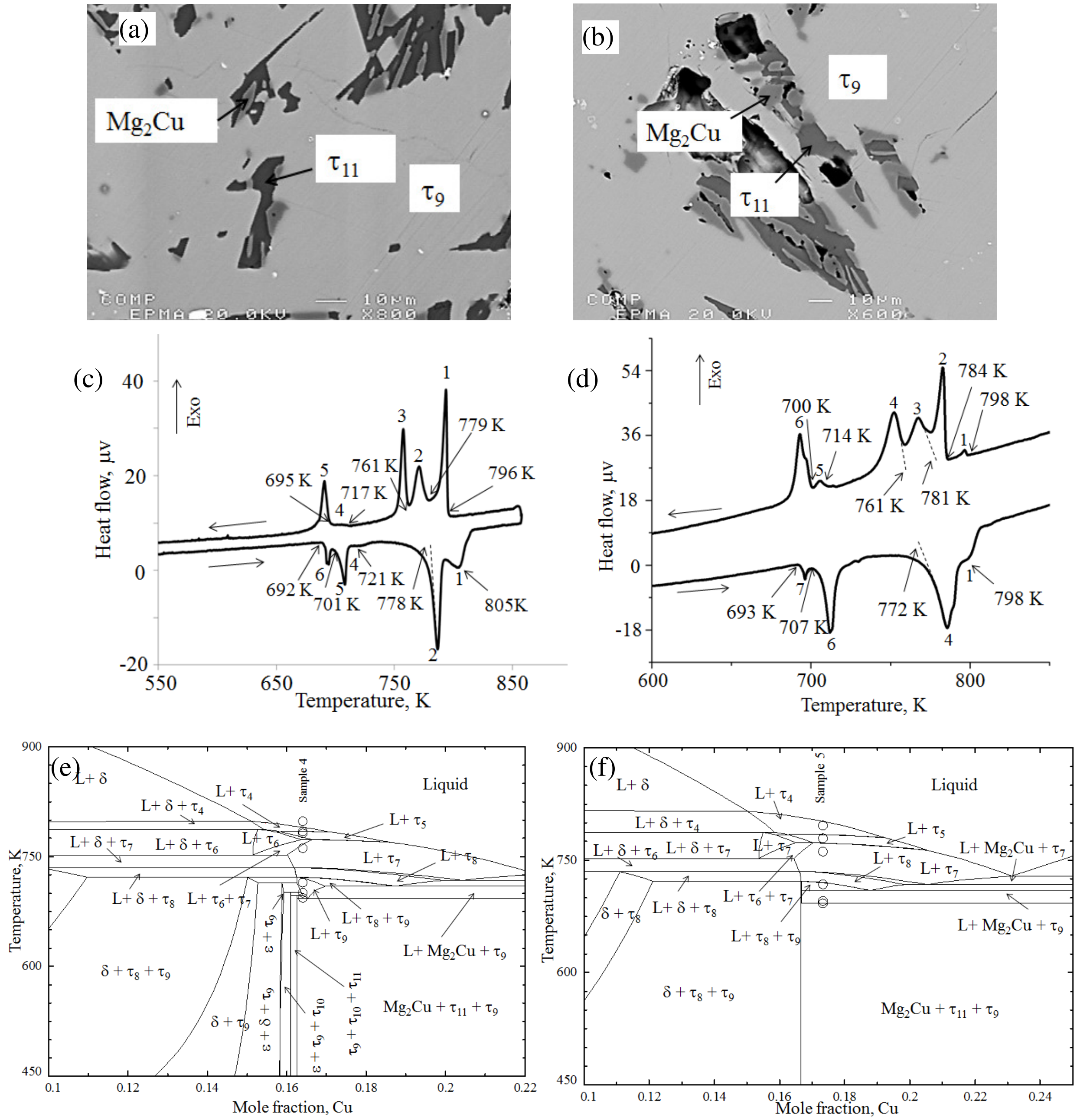

Figure S3: BSE image of (a) sample 4 (67.5/16.4/16.1 Mg/Cu/Y at.%) and (b) sample 5 (66.7/17.3/16.0 Mg/Cu/Y at.%). Both the samples clearly show the three-phase relation between τ_9, τ_{11} and Mg_2Cu. (c) DSC spectra of sample 4 and (d) sample 5. The presence of several arrests in the DSC spectra suggested the occurrence of a rather complicated melting behavior. The complexity arises because of the presence of six ternary intermetallic compounds (τ_4 to τ_9) in close proximity. The corresponding vertical sections at (e) constant 67.5 at.% Mg and (f) constant 66.7 at.% Mg show good agreement with the DSC measurements.

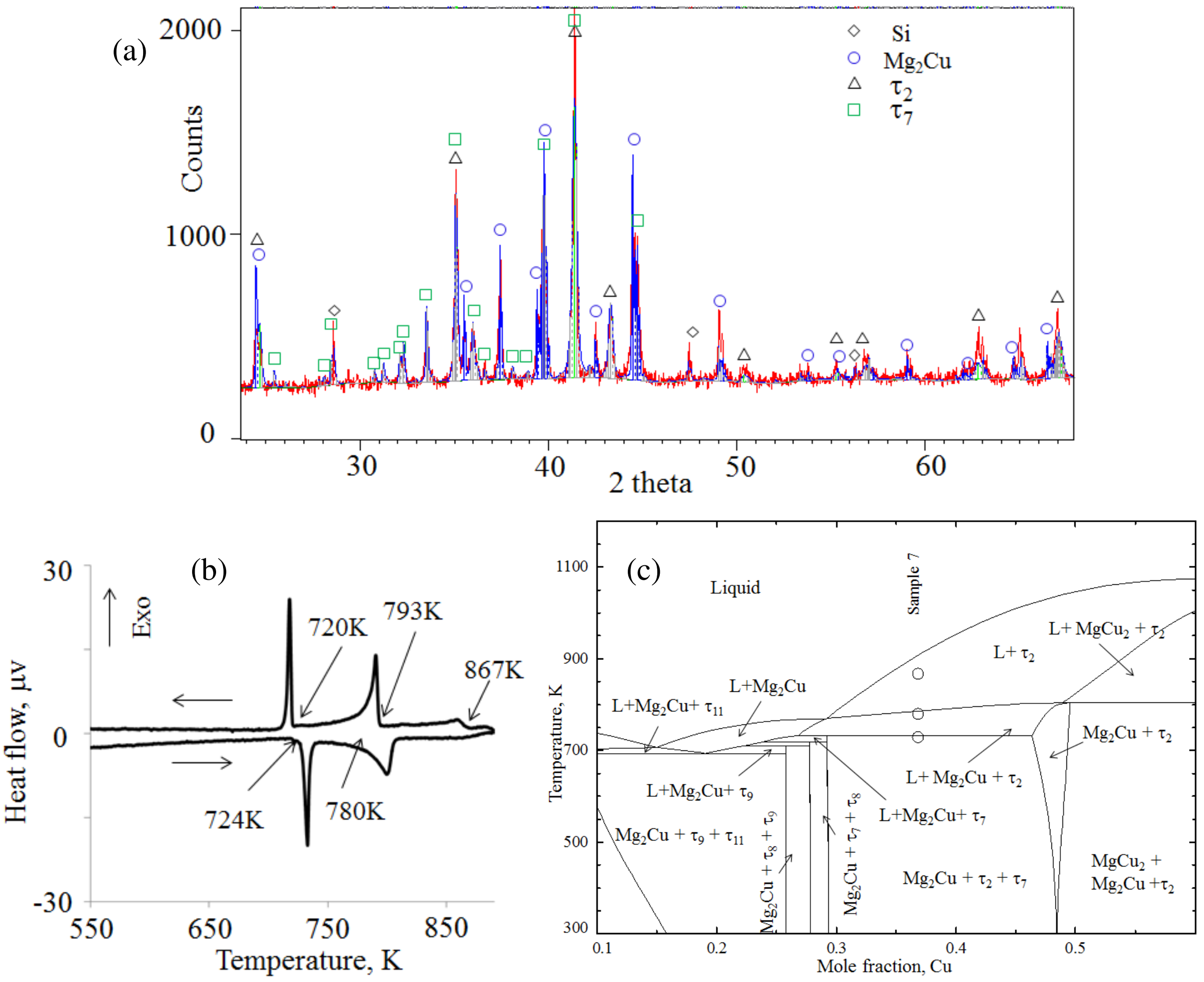

Figure S4: (a) Sample 7 (55.6/36.9/7.5 Mg/Cu/Y at.%) is located in the three-phase region of $Mg_2Cu+\tau_2+\tau_7$. These phases are positively identified in the XRD pattern. (b) DSC spectra and (c) the calculated vertical section at constant 7.5 at.% Y with DSC signals of sample 7.

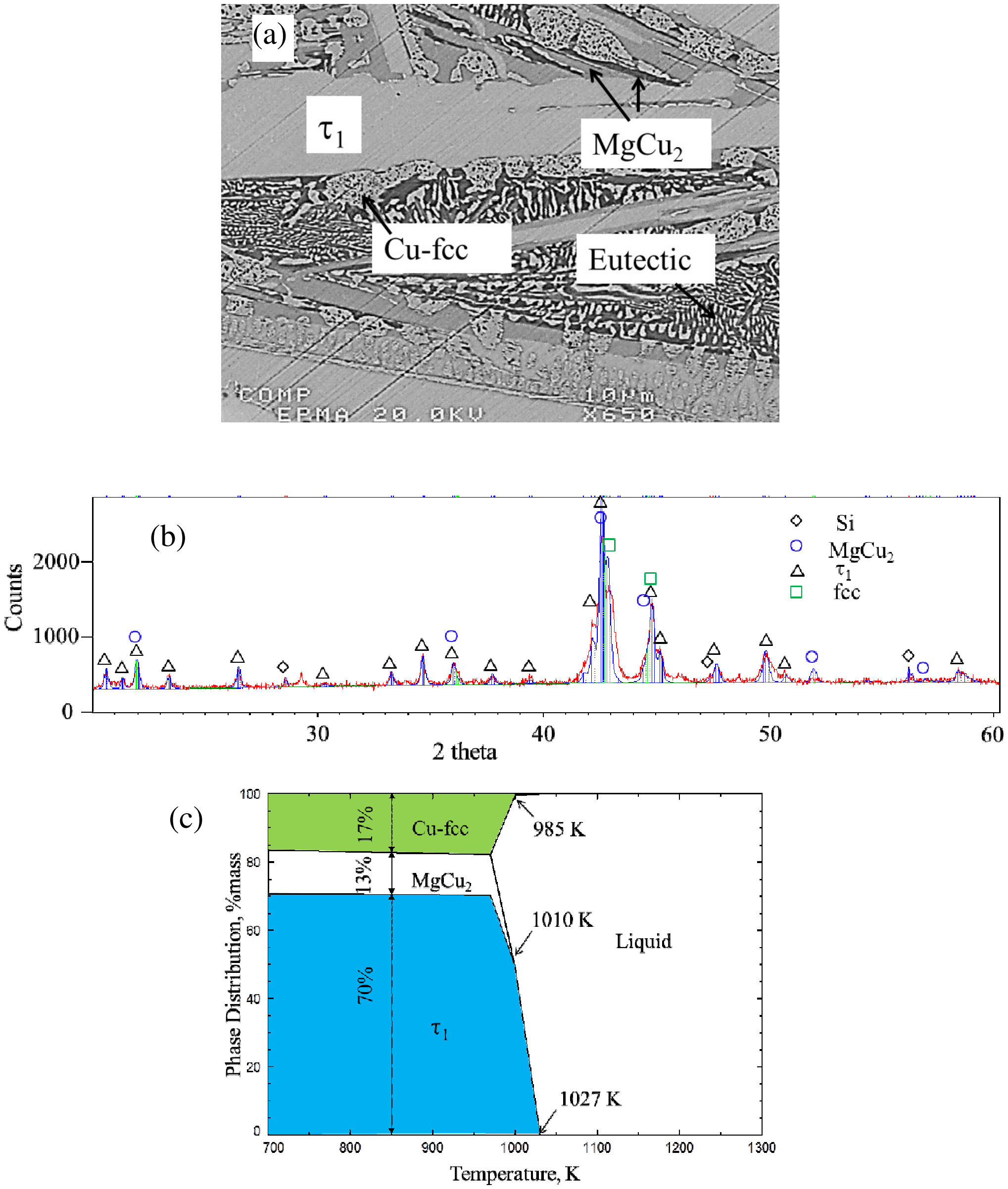

Figure S5: Sample 10 (16.9/77.3/5.8 Mg/Cu/Y at.%) is located in the three phase region of $MgCu_2$+ τ_1+Cu-fcc. The phases are detected in the (a) BSE image and (b) XRD pattern. (c) The calculated phase assemblage diagram of sample 10. According to the thermodynamic calculation, the microstructure of this alloy should contain about 70% τ_1, 13% $MgCu_2$ and 17% Cu-fcc. This is in reasonable agreement with the relative mass fractions of the phases, resulting from Rietveld analysis of the XRD: 63% τ_1, 14% $MgCu_2$ and 23% Cu-fcc.

References

1 Solokha, P. *et al.* Rare earth-copper-magnesium compounds $RECu_9Mg_2$ (RE=Y, La-Nd, Sm-Ho, Yb) with ordered $CeNi_3$-type structure. *Journal of Solid State Chemistry* **179**, 3073-3081 (2006).

2 De Negri, S., Solokha, P., Saccone, A. & Pavlyuk, V. The Y-Cu-Mg system in the 0-66.7at.% Cu concentration range: The isothermal section at 400°C. *Intermetallics* **17**, 614-621 (2009).

3 Mishra, R., Hoffmann, R.-D. & Pottgen, R. New magnesium compounds RE_2Cu_2Mg (RE = Y, La – Nd, Sm, Gd – Tm, Lu) with Mo_2FeB_2 type structure. *Zeitschrift für Naturforschung B, Chemical Science* **56**, 239-244 (2001).

4 Solokha, P., De Negri, S., Pavlyuk, V. & Saccone, A. Inhomogeneous 2D linear intergrowth structures among novel Y-Cu-Mg ternary compounds with yttrium/copper equiatomic ratio. *Solid State Sciences* **11**, 801-811 (2009).

5 Inoue, A., Kato, A., Zhang, T., Kim, S. G. & Masumoto, T. Magnesium-copper-yttrium amorphous alloys with high mechanical strengths produced by a metallic mold casting method. *Materials Transactions, The Japan Institute of Metals and Materials* **32**, 609-616 (1991).

6 Kim, S. G., Inoue, A. & Masumoto, T. High mechanical strengths of magnesium-nickel-yttrium and magnesium-copper-yttrium amorphous alloys with significant supercooled liquid region. *Materials Transactions, The Japan Institute of Metals and Materials* **31**, 929-934 (1990).

7 Institute for Materials Research of Tohoku University. KIND Data Base. (Cited on October 30, 2012). *http://www-db2.imr.tohoku.ac.jp/kind/11_Amor_Ternary/Cu-Mg-Y.html*.
